# Sexual pair-formation in a cicada mediated by acoustic behaviour of females and positive phonotaxis of males

**DOI:** 10.1038/s41598-017-06825-5

**Published:** 2017-07-25

**Authors:** Zehai Hou, Changqing Luo, J. Dale Roberts, Cong Wei

**Affiliations:** 1 Key Laboratory of Plant Protection Resources and Pest Management, Ministry of Education, College of Plant Protection, Northwest A&F University, Yangling, Shaanxi 712100 China; 2 Centre of Excellence in Natural Resource Management, University of Western Australia, Albany, 6330 Australia

## Abstract

The functions of female song found in a few cicadas have rarely been studied. In the cicada *Subpsaltria yangi* we investigated the acoustic behaviour and signal structure of songs produced by females, the phonotaxis of males, and mate choice, as well as the selective pressure imposed on this species by predators. Pair-formation in *S*. *yangi* occurs when males signal, females respond, then males move to signaling females, which is opposite to that in most other cicadas where females move to calling males. Females only mate once and are sexually unreceptive after copulation. Most males mate once, but ~25% mate multiply. Females display little direct evidence of mate preference or choice of males, and all mate encounters led to a successful mating. Only males are attacked by a robber fly, *Philonicus albiceps*, while flying to females. This imposes strong selection on males – only males who can evade predators mate. Males are also attracted to human simulations of female calls. This behaviour exposes the mating system to impacts from anthropogenic noise systems which could disrupt mating activity of this species. Our results improve the understanding of mate choice/competition in cicadas, and are valuable for future studies of the evolution of sound communication in the Cicadoidea.

## Introduction

Acoustic communication is well developed in most species of birds, anurans, and many arachnids and insects^1–5^. Males of most cicada species use a pair of timbals^6, 7^ to produce loud, acoustic signals that are associated with different behavioural contexts such as intrasexual competition, intersexual interactions and defense against predators^8, 9^. Studies of acoustic signals in cicadas have particularly focused on calling songs produced during pair-formation, used by males to elicit acoustic and/or phonotactic responses from conspecific females^10–12^. Calling songs in cicadas are typically species-specific, are essential for mate recognition process^13, 14^, and also function as a reproductive isolating mechanism^15^.

During pair-formation, females of many cicada species are not silent but emit sounds by flicking their wings in response to calling songs produced by conspecific males^16, 17^. The detailed structure and function of female acoustic signals have rarely been investigated.

Some cicada species respond to both conspecific signals and other sounds: as diverse as snapping twigs, hand clapping, finger snaps, the sound of breaking twigs, and as bizarre as lawnmowers, suggesting an ability to discriminate and respond to generalised signal components, e.g. frequency, that may form essential components of intraspecific communication systems and pair formation^18, 19^. The cicada species *Subpsaltria yangi* Chen is unusual in that, besides the conventional timbal organs of males, it has well-developed stridulatory organs that are found in both sexes^20–22^. Pair-formation in *S*. *yangi* occurs when males acoustically signal, females answer advertising males, then males fly to the female signal^21^. This pair-forming system is rare in cicadas, e.g. previously known only in a few cicadas such as the tick-tock cicada *Physeema quadricincta* (Walker)^16, 23^, New Zealand cicadas *Kikihia* spp.^24^, and periodical cicadas *Magicicada* spp.^11^. In contrast, pair-formation in most other cicadas is achieved when responsive females move to calling males^25^. During previous field investigations, we observed that encounters between male and female *S*. *yangi* almost inevitably led to successful matings, that the males displayed unexpected, positive phonotaxis to one type of human vocalizations attempting to imitate female response sounds, and that this species commonly suffered predation by robber flies during pair-formation.

We investigated the structure of acoustic signals and the pair-formation behaviour of females, phonotaxis of males in *S*. *yangi*, and what properties artificial signals that evoked acoustic responses had in common with natural acoustic signals used by this species. Censuses of a natural population were conducted to reveal the preference of females in pair-formation and the number of matings by both sexes. The sex ratio of individuals captured by the predominant predator, the robber fly *Philonicus albiceps* was also investigated to understand the selective pressure imposed on pair-formation of *S*. *yangi* by predators. Our results improve our understanding of mate choice/competition and pair-formation behaviour in cicadas, and will be valuable for future studies of the evolution of sound communication in the Cicadoidea.

## Results

### Sound-producing behaviour of *Subpsaltria yangi* females

**Figure 1:**
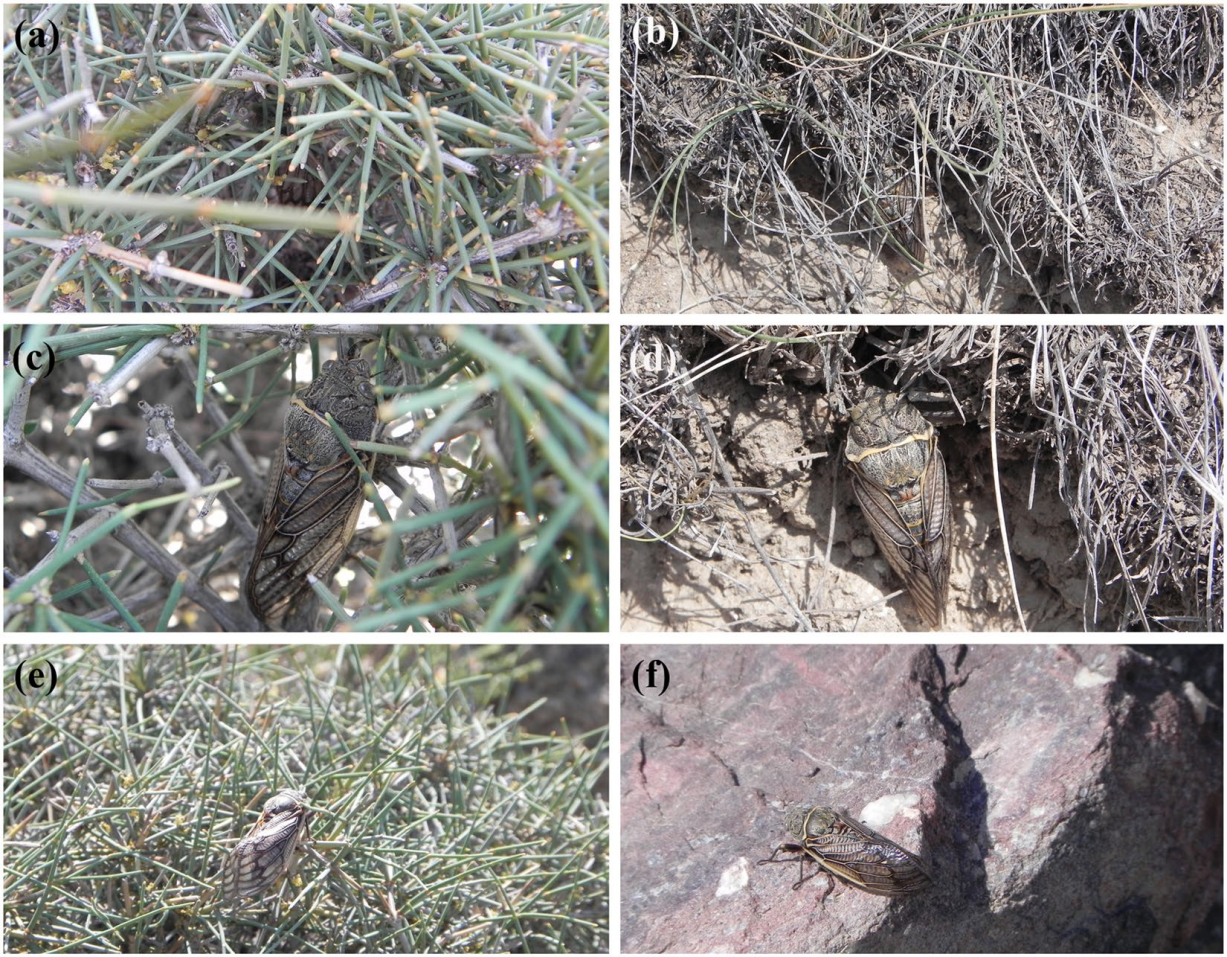
Females and males choose different places to produce sounds. (**a**–**d**) Females. (**e**–**f**) Males. (**a**) A female producing sounds in the canopy of the host plant. (**b**) A female producing sounds among grasses. (**c**) Some stems of the plant were removed to show the signaling female in (**a**), which has the body (including forewings) colours matching to clumps of branches and leaves of the host plant. (**d**) Some leaves of the grasses were removed to show the signaling female in (**b**), which has the body (including wings) colours matching to clumps of the withered vegetation. (**e**) A male calling from exposed perch site (the top of the host plant). (**f**) A male calling on the top of a stone.

Sexually receptive females of *Subpsaltria yangi* emitted sound signals in response to the calling songs of conspecific males. Sounds were generated in association with the upward and downward movements of the forewings (Supplementary Video 1). Females were usually found hidden in the canopy of shrubs and grasses when they produced sounds (Fig. 1a–d). This behaviour was different from that of *S*. *yangi* males which generally emitted sounds in high, exposed perch sites (e.g. the top of plant stems and stones) (Fig. 1e,f). When females were disturbed, they stopped producing sounds and remained immobile on their perches.

### Effect of copulation on sound-producing behaviour of *Subpsaltria yangi* females

Before copulation, all females (N = 12) used in the acoustic playback experiments produced sound signals in response to the calling songs of male *S*. *yangi*. Conversely, none of the mated females (N = 12) responded acoustically to the calling songs. After playback experiments, mated females of this cicada species were kept on their host plants covered with gauze netting for observation. They quickly started to insert their ovipositor in branches of the plants and excavate a series of closely spaced egg nests for oviposition. The female cicadas laid eggs in each nest. The number of eggs in each nest varied from 18–35 (mean ± SD = 26 ± 4.01, N = 18). Mated female cicadas died soon after their last oviposition.

### Acoustic analysis of sounds emitted by *Subpsaltria yangi* females

**Figure 2:**
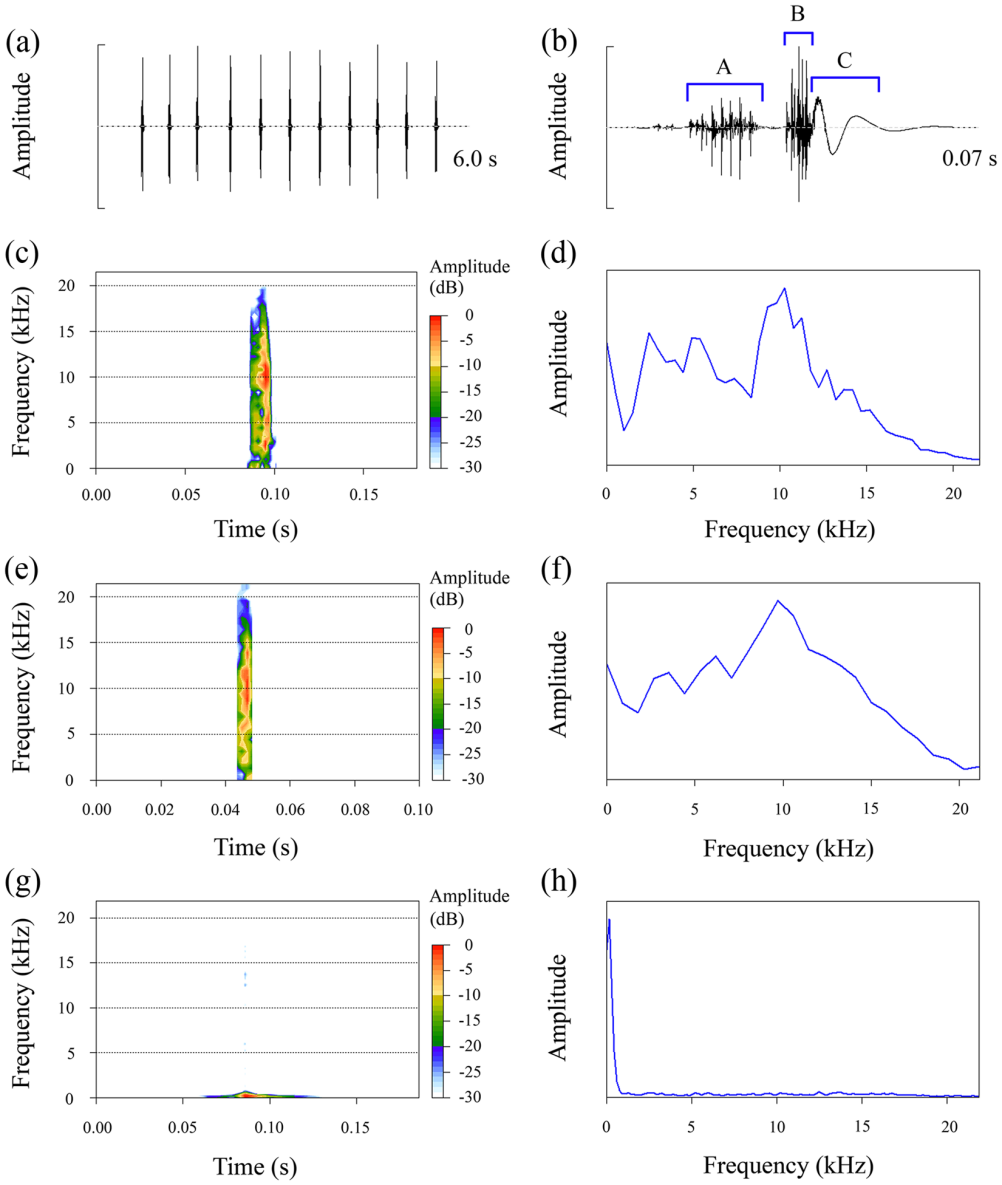
Analysis of sounds produced by female *S*. *yangi*. (**a**) Oscillogram of 11 sounds produced by a female. (**b**) Detailed oscillogram of a single sound. (**c**,**d**) Spectrogram (time *vs* frequency *vs* amplitude, colour amplitude scale given on the right) and mean spectrum (frequency *vs* amplitude) of the component A. (**e**,**f**) Spectrogram and mean spectrum of the component B. (**g**,**h**) Spectrogram and mean spectrum of the component C.

Each movement of the forewings was responsible for producing a single song. A sequence of songs was generated by multiple movements of the forewings (Fig. 2a and Supplementary Video 1). Songs were emitted at a mean rate of 1.53 ± 0.39 per second (N = 116). The interval between songs was highly variable and ranged from 0.19 to 1.94 s (mean ± SD = 0.59 ± 0.30 s, N = 1019).

The structure of a single song was complicated. Each song was composed of three structurally distinct components: component A, component B, and component C^21, 22^ (Fig. 2b). Components A and B were separated by a silent interval, but there was no interval between components B and C (Fig. 2b). Previous study showed that both component A and component B were produced by stridulatory organs, and component C resulted from the impact of the forewings on the body^22^. Temporal characteristics of the songs are summarised in Table 1.

**Table 1:** Temporal characteristics of sounds produced by females of *Subpsaltria yangi*.

Characteristic	*N*	Mean	SD	Min	Max
DA (ms)	1104	16.36	2.77	9	23
DB (ms)	1104	5.38	1.16	3	9
IAB (ms)	1104	4.54	1.31	2	8
DC (ms)	1104	13.20	2.26	5	20

Characteristics: DA, duration of component A; DB, duration of component B; IAB, interval between component A and component B; DC, duration of component C.

Although component A and component B were both produced by the stridulatory organs, there were considerable structural differences between the two song components. For comparison, 1104 songs from more than 20 females were analysed, and the number of pulses was counted in components A and B. Component A was significantly longer than component B (Mann–Whitney U test: Z = −40.888, N_1_ = 1104, N_2_ = 1104, *P* < 0.001; Table 1). The amplitude of component A was relatively lower than that of component B. Both components A and B consisted of a train of pulses. The average number of pulses was 8.01 ± 2.02 (N = 1081) in component A, and 7.30 ± 1.33 pulses (N = 964) in component B. Song recordings where pulse structure was not clear in component A or B, e.g. due to poor recording quality or high levels of background noise, were not included in the analysis. The difference in number of pulses between the two song components was significant (Mann–Whitney U test: Z = −8.268, *P* < 0.001).

Component A and component B were similar in frequency characteristics: both sound components contained a broad spectrum of frequencies, from 1.5 to 20 kHz with the bulk of energy concentrated at about 10 kHz (Fig. 2c,f). Conversely, most of the energy in component C was at very low amplitude and very low frequencies concentrated below 1.5 kHz (Fig. 2g,h).

### Phonotaxis of *Subpsaltria yangi* males to human imitations of female song

We succeeded in attracting *S*. *yangi* males from within a range of about 10 meters by vocalizing to imitate the female response sound, and in some cases, several males were attracted concurrently. By flying from one tree to the next, males approached gradually to the place where the attractive sounds were produced. Finally, males landed on vegetation near us or even on us (Supplementary Video 2). The attracted males were easily captured.

**Figure 3:**
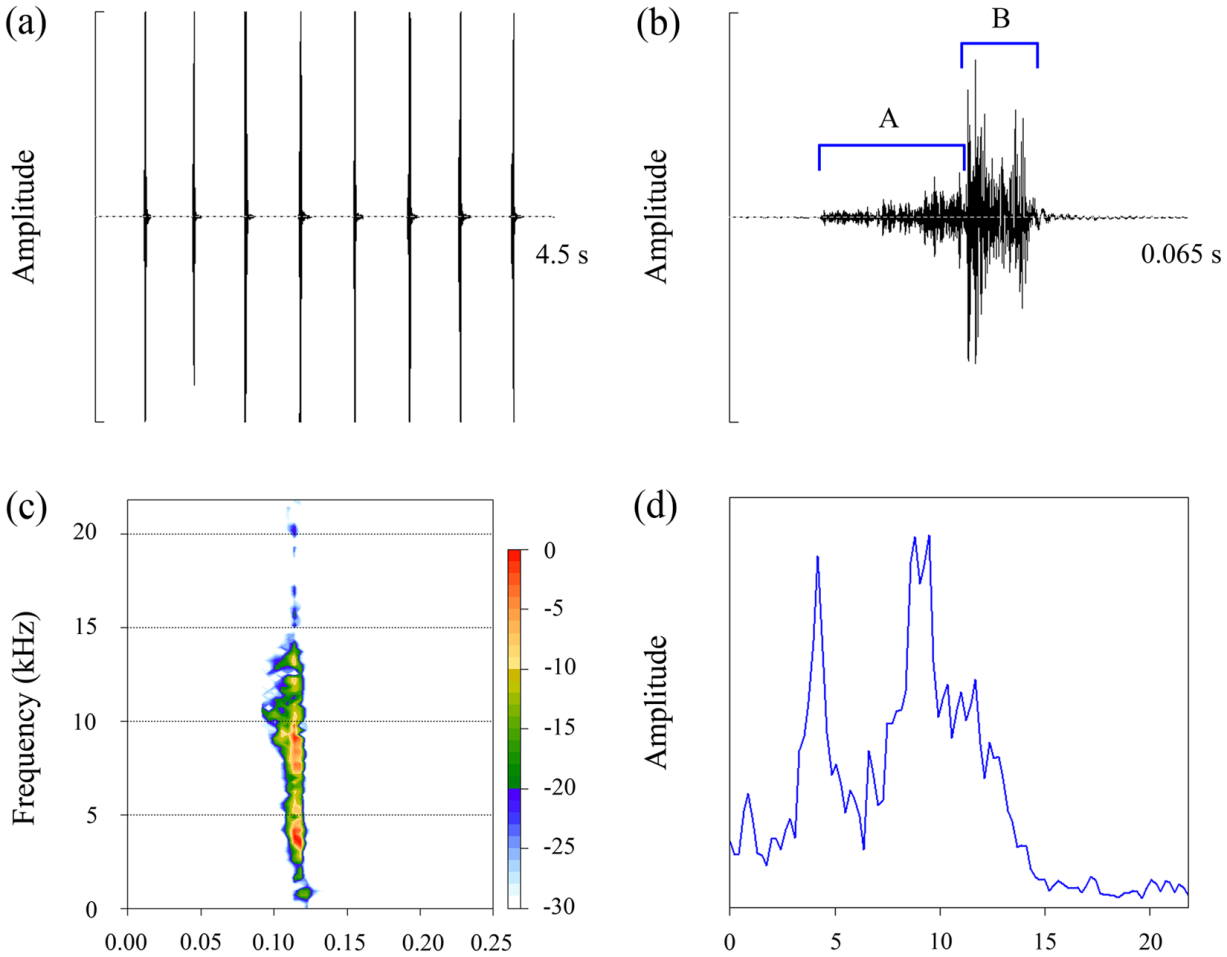
Acoustic structure of the attractive sounds. (**a**) Oscillogram of eight sounds. (**b**) Detailed oscillogram of a single sound. (**c**,**d**) Spectrogram (time *vs* frequency *vs* amplitude, colour amplitude scale given on the right) and mean spectrum (frequency *vs* amplitude) of a single sound.

Oscillograms and spectrograms of the sounds we produced are shown in Fig. 3. The sound was repeated at a rate of 1.89 ± 0.89 (N = 30) sounds per second (Fig. 3a). Individual sound duration was 26.74 ± 3.60 ms (N = 497), and the mean interval between sounds was 0.49 ± 0.087 s (N = 431). Each sound showed a two-component structure (component A and component B) due to a large amplitude modulation (Fig. 3b). The amplitude of component B was higher than that of component A (Fig. 3b). Spectral analysis shows that the major part of sound energy was contained in two frequency bands, i.e. at about 4 kHz, and from 8 to 9 kHz (Fig. 3c,d) comparable to frequency peaks in the natural song (Fig. 2d,f).

**Figure 4:**
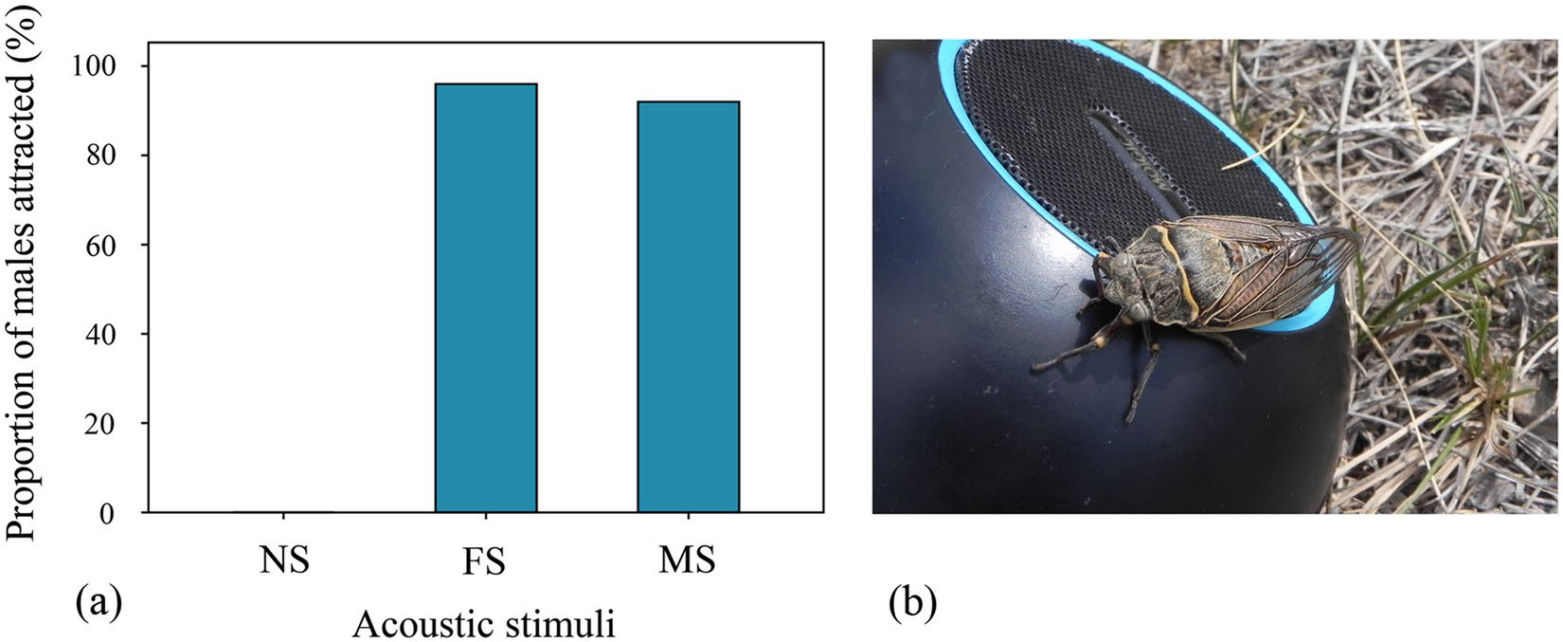
Acoustic playback experiments. (**a**) The efficiency of the playback stimuli in eliciting phonotactic responses from male cicadas. FS: the sounds produced by *S*. *yangi* females; MS: the sounds produced by an observer’s mouth; NS: no sounds. (**b**) A male was attracted by playback of MS and landed on the loudspeaker.

Acoustic playback experiments showed that our vocal sounds can elicit strong positive phonotactic responses from calling males of *S*. *yangi* (N = 25). This type of sound was as effective as the sounds produced by *S*. *yangi* females in attracting male cicadas, i.e. 23 were attracted by our vocal sounds, and 24 attracted by the latter (Fisher’s exact test: *P* > 0.05; Fig. 4a). The males flew directly towards the loudspeaker that was broadcasting acoustic stimuli. They landed near or even on the loudspeaker (Fig. 4b). In addition, we found that some attracted males had their genitalia extruded.

### Mating frequency in *S*. *yangi*

**Figure 5:**
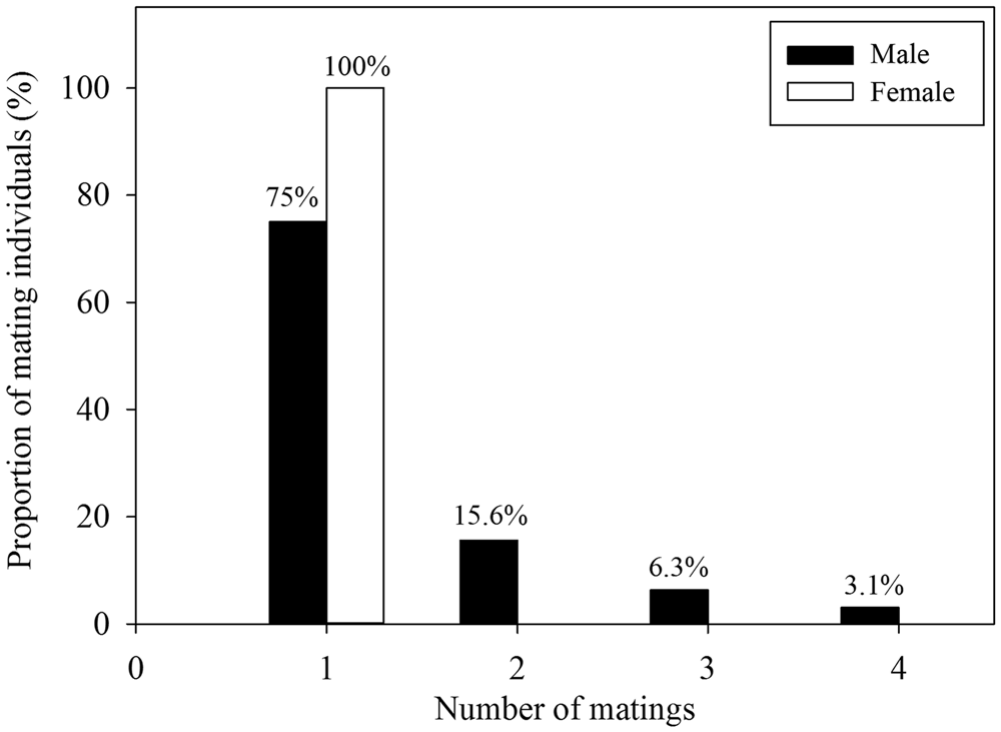
Mating frequencies of males and females of *S*. *yangi*.

In the 2015 breeding season, we found that 32 males mated with 44 females in our censuses of a natural population based on capture-mark-recapture/resight (CMR) data. All 44 females mated only once and then became sexually unreceptive after mating (Fig. 5). They all mated with the male which flew towards and landed first in the vicinity of the responding female during an acoustic exchange. They did not engage in mate search after mating. Mated females remained, immobile, on their perches after copulation for ~48 h, and then started to excavate egg nests in branches of the host plants. Although most males (24 out of 32 males, 75%) mated only once, some males mated twice (15.6%), three (6.3%), or even four times (3.1%) with different females (Fig. 5). Females of *S*. *yangi* are strictly monandrous, but about 25% of males mate multiply.

### Sex-biased predation on *Subpsaltria yangi* males

During field investigations in 2015, we captured one robber fly (*P*. *albiceps*) eating a male *S*. *yangi*. In 2016, 14 robber flies that had captured a male cicada were collected. No robber flies ever captured female cicada: predation on cicadas was strongly male-biased (χ^2^ = 13.07, *P* < 0.001).

**Figure 6:**
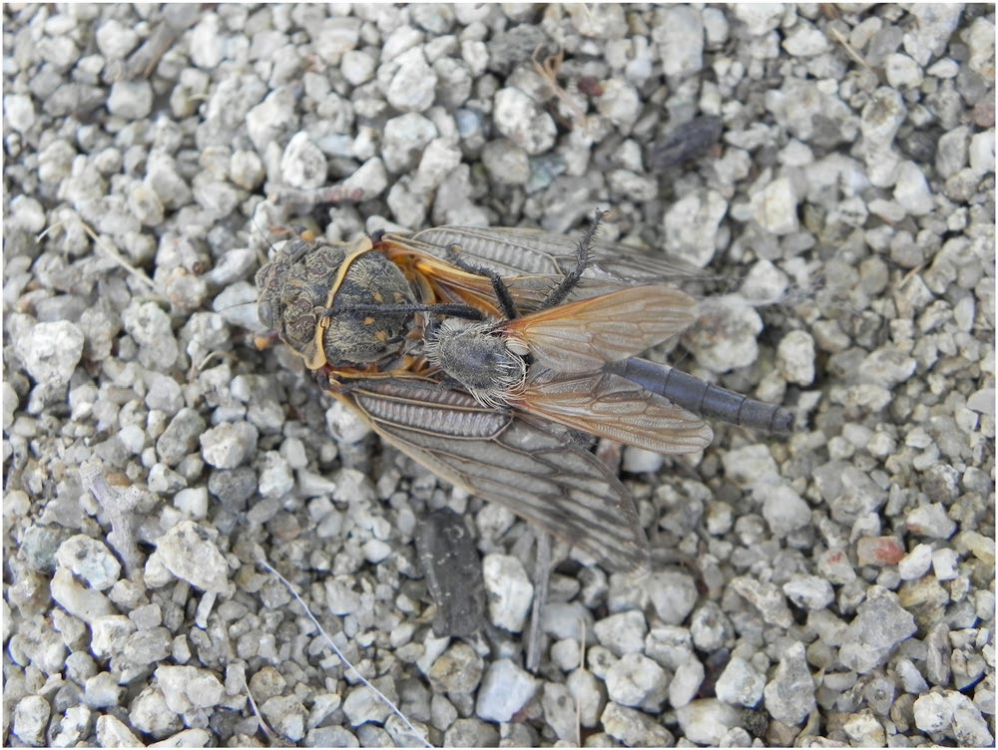
A male of *S*. *yangi* captured by a robber fly: *Philonicus albiceps*.

Male cicadas were all attacked in flight, especially in their call-and-fly mate-seeking activity. The robber flies attacked *S*. *yangi* suddenly, capturing the cicada with their strong legs, and piercing the cicada body with the well-developed proboscis (Fig. 6 and Supplementary Video 3). In our playback experiments of male song to females, and in 25 playbacks of female cicada calls and human simulations of female cicada calls, respectively, robber flies were never attracted to playback of cicada calls (playback details above) suggesting they are not orienting to acoustic signals of cicadas of either sex but are likely visual predators.

## Discussion

Pair-formation in *S*. *yangi* differs from that of most other cicadas in that females of this species acoustically answer advertising males and males move to signaling females^21^. This pair-formation pattern is the same to that of the tick-tock cicada *Physeema quadricincta* ^16, 23^, New Zealand cicadas *Kikihia* spp.^24^, and periodical cicadas *Magicicada* spp.^11^ where the female sound is generated by a single wing flick and consists of only one component^16^. However, females’ signals in *S*. *yangi* seem more complex due to the stridulatory organs involved, with each song comprising three structurally distinct components. In contrast, pair-formation in most other cicadas is achieved when responsive females move to calling males^25^. Previous studies have shown that the sounds produced by females of *S*. *yangi* (i.e. female sexual signals) convey information about the species identity, sex, receptive state and location of the female cicadas, and the information can be used by conspecific males to recognize and find females^21^. We have established that female *S*. *yangi* become sexually unreceptive after copulation: females only mate once, which is similar to female cicadas of the genus *Magicicada* ^11, 26^. Our field observation suggests that all encounters between male and female *S*. *yangi* led to a successful mating and no further mating by females: females are monogamous. Previous studies showed that in some species monandry may be imposed on females by males, e.g. males may physically block the female reproductive tract using mating plugs, manipulate female physiology to reduce female receptivity or deposit chemicals which make females unattractive to rival males^27^. Female cicadas in many species receive a seminal plug after copulating and probably only mate once, while males may mate several times^26, 28^. Given that *S*. *yangi* males make females permanently unreceptive following mating, monandry in this species may be imposed on females by male manipulation. Mating success is not the only contributor to reproductive success, which depends also on naturally selected fitness components such as fecundity, or compatability^29^. About 25% of *S*. *yangi* males mated two or more times, and mated males might be sperm depleted. We do not know whether mating with an already mated male affects fertilization success or the fitness of the offspring. The biology of *S*. *yangi* indicates this species is open to experimental manipulation, and exploration of further aspects of how sexual selection affects mating patterns and success in this species is desirable.

Behavioural observations showed that females of *S*. *yangi* prefer to produce sounds from concealed perches, but do not move to advertising males contrary to the pattern in most other cicada species. Predation may have shaped this novel calling system. Communication systems using visual, chemical, and acoustic signals are prone to exploitation by predators and parasitoids^30–34^ that may impose a strong selective pressure on sound emitters. In this system robber flies catch flying males, and Eurasian magpies have been observed catching and eating, or, suppressing acoustic signaling by females. But there is no evidence predators are using any component of the acoustic signals to locate cicadas of either sex. In field investigations we found *S*. *yangi* females tended to fall silent and remained still on their perches when facing a predator such as a Eurasian magpie or a human.

Unlike females, males of *S*. *yangi* produced sound signals from high, exposed perch sites. They flew frequently from perch to perch, making many short flights when responding to receptive females. Gwynne^16^ described a similar risky, call-and-fly mate-seeking activity in the male tick-tock cicada *Physeema quadricincta*, which led to males being captured more often than females by web-building spiders: exactly comparable to the sex biased predation by robber flies we observed. The call-and-fly mate-seeking activity allows males to signal and listen for the response sounds of females over a larger area than could be achieved with sedentary calling.

Behavioural observations, acoustic playback experiments and censuses focused on matings in the present study all suggest that females of *S*. *yangi* mate once. Females are likely to constitute a limiting resource for males, i.e. the operational sex ratio is highly skewed toward males, which results in intense male-male competition for mating opportunities^35–37^. Sexual selection (e.g. male-male competition) in males favors male traits (e.g. elaborate physical ornaments and conspicuous displays) that lead to high reproductive success, even at the cost of increased mortality^38, 39^. Our results show that *S*. *yangi* males but no females suffered predation by the robber fly *P*. *albiceps*. This is a case of strongly sex-biased predation which results from the behaviour of males: their flight to females. The selective advantage of risk-taking by competing males comes from increased opportunities to inseminate females^40^. Therefore, although the call-and-fly mate-seeking behaviour may increase conspicuousness of the *S*. *yangi* males to natural enemies, selection may favor this form of signaling behaviour since singing from high and exposed locations can increase the efficiency of signal transmission and the range of communication, which increases a male’s probability of obtaining females^41^. However, this pair-formation system does put mate-seeking males at greater risk of predation than females. Males of *S*. *yangi* displayed highly sensitive responses to female sexual signals, but no evidence of male mate choice was found amongst females (i.e. all encounters between male and female led to a successful mating). Predation pressure may have shaped this ‘love-at-first-sight’ – immediate pair-formation. The male call and female call in response may have two benefits for females: a) sampling calls from several males may allow selectivity of response based on male call attributes or related male quality; and b) male movement and exposure to predation risk reduces predation risk to females. Females respond to calls from an array of calling males and the female signal response may be timed to better target particular males: e.g. males with particular frequency properties (possibly reflecting body size), or calling rates (stamina) that reflect male fitness. If the sound field around females is directional, they may orient body position to optimize transmission to particular males. These are attributes that are known in frogs and insects, and known to influence mating success in frogs and insects^3^. However, its influence on cicadas remains unknown. Our results indicate that the robber fly *P*. *albiceps* is a visual predator. This visual mechanism has also been revealed in a tiny robber fly, *Holcocephala fusca*, in recent studies^42, 43^. The call-and-fly mate-seeking by males reduces the risk of females being captured by aerial predators but clearly does not eliminate that risk for males. Predation on flying males may generate indirect female choice: mated males have evaded aerial predators – a property that may be advantageous to offspring of either sex. Females might also exert indirect choice if first males to arrive have better call locating abilities or faster flying speeds^44, 45^. The successful playback experiments reported here indicate this species is open to experimental manipulation to test for call preference in females in the future.

Our previous study has revealed that, although the acoustic response signals of *S*. *yangi* females are composed of stridulation (component A + component B) and impact sounds (component C), only stridulation played a role in intraspecific communication^22^. Here, we found that a vocal sound produced by humans can also be effective in eliciting searching and courtship behaviour in males of this cicada species. The frequency structure of acoustic signals of females and the human imitations are broadly similar: both have a marked frequency peak at ~10 kHz. The human imitation does not replicate amplitude modulation (pulsing) patterns in parts A and B of female signals, or the very low frequency and low amplitude sounds in part C of female signals, suggesting frequency components are sufficient to elicit flight responses by males.

Similar to the males of *S*. *yangi*, males of the mute cicada *Karenia chama* Wei & Zhang can be easily attracted to sounds produced by clapping of hands, knocking of bamboo sticks and breaking of twigs^18^; a few other cicada species can be attracted by sounds of lawnmowers and finger-snaps^18, 19^. The most likely explanation for the phonotaxis of these cicadas is that males mistakenly identify the artificial sounds as acoustic response signals of conspecific females.

The cicada *S*. *yangi* is endemic to China and is the sole species of the subfamily Tettigadinae known from China^20^. It has a restricted distribution from the Loess Plateau and adjacent areas^21^. Fragmentation of remaining habitats due to human activities is a critical threat to populations of this species. Our acoustic playback experiments showed that mated females of *S*. *yangi* that were kept on their host plants covered with gauze netting quickly started to excavate egg nests for oviposition; but mated females under natural conditions remained still on their perches after copulation for around 48 h, and then started to lay eggs. We presume this indicates some risk to females during oviposition, e.g. from predators. The phonotaxis of *S*. *yangi* males also makes this species susceptible to negative impacts of anthropogenic sounds which might disrupt mating activity of this species^46, 47^. Anthropogenic noise produced by human activities affects acoustic communication in the cicada *Cryptotympana takasagona* in the Kaohsiung metropolitan areas of southern Taiwan, which did not call more in noise gaps and acoustic features of its calling songs significantly increased with ambient noise levels^46^. However, it was also revealed that there was no significant temporal partitioning of cicada assemblages in either noisy city or natural mountain environments, and that for the common species found in both environments, the calling activity patterns at both seasonal and diel time scales were significantly consistent across sites and across environments^47^. Future exploration of whether the normal mating patterns or resource costs of obtaining mates of *S*. *yangi* may be affected by anthropogenic sounds and/or other abiotic stimuli is desirable. The results may be helpful for the conservation of this rare cicada species.

The results presented here are a valuable contribution to understanding patterns of sexual selection and mate choice in cicada species with the rare pair-formation system that is achieved when the males fly to signaling females, and a contribution to understanding tactics of pair-formation, sexual selection, and mate choice in other animal species which suffer selective pressure from predators or parasitoids. We suggest that studies focusing on acoustic behaviour and signal structure of female cicadas are needed, and information obtained from these acoustic studies would provide important insights into the evolution of acoustic communication system of this group of insects.

## Methods

### Species and study site

*Subpsaltria yangi*, a medium-sized cicada (body length 28.00–33.20 mm) described in 1943, is the only known species of the subfamily Tettigadinae in China. Live specimens of the insect had not been seen in the field since 1989, but in June 2011, we found a large population of *S*. *yangi* in Chunshugou valley (38°33.699′N, 105°55.217′E) located in the Helanshan National Nature Reserve, Ningxia Hui Nationality Autonomous Region, China, occurring mainly at elevations of 1400–1600 m above sea level. Vegetation in the dry habitat occupied by *S*. *yangi* consists primarily of drought-tolerant dwarf shrubs and herbaceous plants. Adults of this cicada species feed mainly on *Ephedra lepidosperma* (Ephedraceae) which is the dominant plant species in this habitat. Field investigation and behavioural observations were conducted in late May and June, 2014–2016, acoustic playback experiments in 2014, and censuses primarily in 2015 and 2016.

### Sound recording and analysis

Acoustic recordings were made using a digital recorder with in-built stereo microphones (PCM-D50, Sony, China; frequency range 20–20000 Hz, 44.1 kHz/16 bit sampling resolution). The distance between the microphone and the insect was between 10 and 20 cm. Sounds were recorded in WAV file format, and sounds recorded on the left channel were used for acoustic analysis. The sounds were edited and analysed using Raven Pro 1.4 (The Cornell Lab of Ornithology, Ithaca, NY, USA) and the Seewave package^48^, a custom-made library of the R software platform^49^. The temporal properties of the sounds were measured from oscillograms, and the frequency parameters were obtained from spectrograms. Only high-quality sounds (low background noise, no overlap with other sounds) were included in acoustic analyses. The terminology used to describe acoustic signals follows that of Alexander^1^. All acoustic recordings were made between 9:00 and 15:00 hours, a period that corresponded to the peak acoustic activity of this cicada species. In reporting analysis of song structure we combined data from all analysed calls from all recorded individuals. We recognise this may bias the data if some individuals have more songs analysed than others, but the broad pattern of song structure was constant across all individuals within sexes, so we anticipate that if this error exists it is small.

### Effect of copulation on sound-producing behaviour of *Subpsaltria yangi* females

Previous studies have shown that sexually receptive females of *S*. *yangi* produced sounds in response to calling songs (i.e. advertisement signals) emitted by conspecific males, and the male cicadas relied on the female sound signals to locate and find the responding females^50, 51^. Our preliminary observations suggested that once mated, females of this cicada species stopped emitting sound signals in response to advertising males. To experimentally determine whether females who had copulated still produced sounds in response to calling songs of males, we conducted acoustic playback experiments in the field. Calling songs from male *S*. *yangi* that were effective in eliciting acoustic responses from females were recorded and played back to females. Acoustic stimuli that consisted of 29 alternating timbal and stridulatory sounds, lasting for 14.7 s, were repeatedly played back using a Sony PCM-D50 Linear PCM Recorder and a Mogic Q2 loudspeaker (frequency response, 150–20000 Hz). We used a digital sound level meter (GM1357, Benetech; fast response, A weighting) to measure sound pressure levels. The peak output intensity of the loudspeaker was adjusted to the natural levels, 85 dB SPL measured at 50 cm from the loudspeaker.

Males and females were collected from their natural habitat before playback experiments were performed. Only males that were producing calling songs and females that were emitting sound signals in response to calling songs of males were captured and used in experiments. Once collected, males and females were kept separately on their host plants covered with gauze netting. Females were tested individually. A female was placed in a cage (0.4 × 0.3 × 0.3 m) and allowed to adapt to the ambient conditions for 3 minutes. Calling songs of male *S*. *yangi* were then repeatedly played back from the loudspeaker placed about 1 m from the female. The acoustic stimuli were presented for 3 minutes. After this playback test, a male was introduced into the same cage. When the cicadas successfully completed copulation, the calling songs were played back to the female for a further 3 minutes. During each of the playback periods, we recorded whether the female emitted sounds in response to the playback.

### Phonotaxis of *Subpsaltria yangi* males

During our field investigation, we accidentally discovered that males of this cicada species displayed positive phonotaxis to human vocalisations. The amplitude, temporal and spectral features of this type of sound were analysed.

We conducted acoustic playback experiments in the field to compare the efficiency of the two types of sounds: (a) produced by *S*. *yangi* females, and (b) human vocalisations in attracting males of this cicada species. Two high-quality acoustic recordings (i.e. having high amplitude relative to background noise and no overlap with other sounds) obtained respectively from one female of *S*. *yangi* and the sound produced by an observer were used as playback stimuli. The playback equipment used was similar to that described above. Prior to each playback, the intensity of the stimulus was adjusted to the natural levels of female sound production, 60 dB SPL measured at 50 cm from the loudspeaker.

Twenty-five males were used for each of the two types of playback stimuli, and no cicada was tested more than once with any one experimental stimulus. In addition, 25 males were used in control playback experiments in which no acoustic stimulus was emitted by the loudspeaker. Therefore, 75 playback experiments were carried out with 75 different male cicadas. For each playback test, an actively signaling male was located in its natural habitat and the loudspeaker was placed on the ground about 3 m from the male. Then, one of the two acoustic stimuli or no stimulus was presented from the loudspeaker. During the test, we recorded whether the male responded phonotactically to the loudspeaker. A phonotactic response was noted if the male flew towards and landed within 50 cm of the loudspeaker. Males who flew away or remained still after 3 min of the playback were considered to have made no positive phonotactic response. After each test, we moved to another site and carried out a new playback test with a different calling male. All acoustic playback experiments were conducted between 9:00 and 15:00 hours.

### Censusing procedure to estimate mating preference and frequency in both sexes of *Subpsaltria yangi*

Censuses of a natural population of *S*. *yangi* in Chunshugou valley of the Helanshan National Nature Reserve were conducted using capture-mark-recapture/resight from 23 May to 22 June 2015. We investigated the preference of females in pair-formation and the number of matings in both sexes. Virgin adults of *S*. *yangi* were captured and marked with water-proof red colour (Veiao Co. Ltd., China) on their right anterior wing with a unique combination of three, easily readable, digits after their emergence. Capturing and marking were carried out in the field and animals were immediately released at the same location. We performed surveys on a daily basis between 9:00 and 15:00 hours, the time period coinciding with sustained chorus activity. During the surveys, whenever a pair of mating cicadas was observed, we noted the three digit identifier of both males and females. If a copulating male or female was unmarked, we captured the pair and marked it after the mating.

### Sex ratio of *Subpsaltria yangi* captured by the robber fly *Philonicus albiceps*

Individuals of *S*. *yangi* suffering predation by the robber fly *P*. *albiceps* were observed during field investigations in 2015 and 2016. The sex ratio of *S*. *yangi* captured by *P*. *albiceps* was investigated in an area of ~0.2 ha in 2016. All observations were carried out in daytime, with different start and ending points every day to avoid any bias due to time of investigation. When an individual *S*. *yangi* was found attacked by a robber fly, we captured the fly and the cicada together using a sweep net, and the sex of cicada was determined by visual inspection.

### Statistical analysis

Statistical analysis was undertaken with SPSS 17.0 software (IBM, Armonk, NY, USA). Data are presented as means ± SD. All statistical tests were two-tailed, and *P* < 0.05 was considered significant.

### Ethical note

This study was carried out in full compliance with the laws of the People’s Republic of China. No specific permits were required for our field investigation. The study species is not included in the ‘List of Protected Animals in China’.

## Electronic supplementary material

Supplementary Information

Supplementary video 1

Supplementary video 2

Supplementary video 3
