# Supplementary material for: Sexual pair-formation in a cicada mediated by acoustic behaviour of females and positive phonotaxis of males
Source: Sci Rep. 2017 Jul 25;7:6453. doi: 10.1038/s41598-017-06825-5 (PMC5526892; doi:10.1038/s41598-017-06825-5)
Supplement: Supplementary file 1 — Supplementary Information [file 41598_2017_6825_MOESM1_ESM.doc]

**Video legends**

**Supplementary video 1: A female of *Subpsaltria yangi* produces sounds in response to the calling songs of a male**

**Supplementary video 2: A male of *Subpsaltria yangi* is attracted by the sounds produced by the mouth of an observer**

**Supplementary video 3: A robber fly of *Philonicus albiceps* is attempting to capture a male of *Subpsaltria yangi***
